# Immunoglobulin A and liver diseases

**DOI:** 10.1007/s00535-017-1400-8

**Published:** 2017-10-26

**Authors:** Tatsuo Inamine, Bernd Schnabl

**Affiliations:** 1 Department of Pharmacotherapeutics, Nagasaki University Graduate School of Biomedical Sciences, 1-7-1 Sakamoto, Nagasaki, 852-8102 Japan; 2 Department of Medicine, University of California, San Diego, MC0063, 9500 Gilman Drive, La Jolla, San Diego, CA 92093 USA; 3 Department of Medicine, VA San Diego Healthcare System, San Diego, CA 92161 USA

**Keywords:** IgA, ALD, NAFLD, NASH, Gut microbiome

## Abstract

Immunoglobulin A (IgA) is a major immunoglobulin isotype in the gut and plays a role in maintenance of gut homeostasis. Secretory IgA (SIgA) has multiple functions in the gut, such as to regulate microbiota composition, to protect intestinal epithelium from pathogenic microorganisms, and to help for immune-system development. The liver is the front-line organ that receives gut-derived products through the portal vein, implying that the liver could be severely affected by a disrupted intestinal homeostasis. Indeed, some liver diseases like alcoholic liver disease are associated with an altered composition of gut microbiota and increased blood endotoxin levels. Therefore, deficiency of SIgA function appears as a significant factor for the pathogenesis of liver diseases associated with altered gut microbiome. In this review, we describe SIgA functions on the gut microbiome and discuss the role of IgA for liver diseases, especially alcoholic liver disease and non-alcoholic fatty liver disease/non-alcoholic steatohepatitis.

## Introduction

The human gut microbiota, comprised of 100 trillion bacteria with a high diversity, has established a mutualistic relationship with their host. While hosts provide a nutrient-rich environment, bacteria metabolize indigested foods, produce beneficial products like vitamins, and educate our immune system. Although the gut microbiota homeostasis is tightly regulated by both host and microbiota themselves, deviation from this highly balanced condition influences our health. A disrupted intestinal homeostasis is not only associated with gut disorders like inflammatory bowel diseases [1, 2] but also with extraintestinal manifestations such as obesity [3, 4], diabetes [5, 6], autism spectrum disorder [7, 8], and liver diseases [9–11].

To maintain the homeostasis, the gut mucosa releases anti-microbial peptides and secretory immunoglobulins [12]. Immunoglobulin A (IgA) is a major immunoglobulin isotype in the gut and several grams of IgA are secreted into the intestine each day in humans [13]. Plasma cells in the intestinal lamina propria secrete IgA, which is transcytosed across intestinal epithelial cells by a cellular receptor, polymeric-immunoglobulin receptor (pIgR) [14]. In the gut lumen, secretory IgA controls microbiota composition and also serves as the first-line barrier that binds bacteria, limits contact between bacteria and enterocytes to prevent bacterial invasion [14–16]. Mice that lack functional IgA in the gut showed altered microbial composition and increased susceptibility to infectious diseases [14, 17, 18]. In addition, mice carrying a genetic mutation in activation-induced cytidine deaminase, which cannot undergo somatic hyper mutation and class switching to IgA and IgG, have bacterial translocation and intestinal bacterial overgrowth [19].

The liver is the front-line organ that receives and responds to gut-derived products through the portal vein, implying that the liver could be severely affected by the disrupted interaction between host and gut microbiota. Indeed, pattern-recognition receptors on Kupffer cells respond to gut-derived bacterial components like lipopolysaccharide (LPS) and contribute to local inflammation [20]. Conversely, the liver also affects gut microbiota through secretion of bile, which contains bile acids and liver-derived IgA, into the intestine. A disrupted homeostasis in the gut has been reported to be associated with chronic liver diseases including primary sclerosing cholangitis, liver cirrhosis, alcoholic liver disease (ALD), and non-alcoholic fatty liver disease/non-alcoholic steatohepatitis (NAFLD/NASH) [21, 22]. In this review, we will describe the IgA function on the gut microbiome and gut–liver axis, and discuss the potential role of IgA for liver diseases, especially ALD and NAFLD/NASH.

## Secretory immunoglobulin A in the gut

### Source and function of IgA

In the intestine, naïve B cells are found in the lamina propria immediately underlying the epithelium and are clustered in the gut-associated lymphoid follicles, such as the Peyer’s patches (PP). By presentation of bacterial antigens and aids from T cells in the germinal center of the gut-associated lymphoid follicles, naïve B cells are activated and become IgA-producing plasma cells. In addition, activation of B cells was found to also occur independently from T cells and specific antigens. In general, T cell-dependent IgA production is related to higher specificity and affinity to bacterial antigens than T cell-independent IgA [23]. There are several extensive reviews about this B cell activation and IgA production process in the gut [24, 25].

IgA-producing plasma cells translocate to the lamina propria and work as a main source of the gut IgA. In the lamina propria, plasma cells produce predominantly dimeric IgA and the dimeric IgA is transcytosed across gut epithelium by a membrane receptor, pIgR that is expressed on the basolateral surface of epithelial cells. At the apical surface of epithelial cells, the IgA-pIgR complex is cleaved and IgA bound to an outer membrane part of pIgR, which is known as secretory component (SC), is released as a secretory IgA (SIgA) [14]. Although SIgA produced in the lamina propria constitutes a major part of total IgA in the adult gut, there are two other sources of gut IgA: maternal milk IgA and liver-derived IgA. The milk SIgA is the only source of IgA in the gut of newborn mammals and contributes to the composition of the first gut microbiome [26], instead of endogenous SIgA that starts to be produced several months after birth in humans and slowly increases [27, 28]. On the other hand, liver-derived IgA is produced by plasma cells that colonize portal regions and the submucosa of biliary tracts, and is secreted to bile by pIgR on hepatocytes and cholangiocytes [29, 30]. In the human liver, however, pIgR is expressed only on cholangiocytes [29]. The proportion of the liver-derived SIgA to total gut SIgA varies between species: low in human and dog, moderate in mouse, and high in rabbit and rat [29]. There is a comprehensive review discussing liver IgA [29].

Although precise mechanisms how SIgA maintains gut homeostasis and protects the host from pathogenic stimuli of bacteria are not known, several mechanisms have been suggested [14, 15]. SIgA prevents the access of bacteria to the apical surface of epithelial cells by agglutinating bacteria and/or entrapping bacteria in the mucus layer. Moreover, binding of immunoglobulins can alter gene expression of some bacteria to reduce their virulence or motility [31, 32]. In addition to direct effects on bacteria, SIgA neutralizes bacterial toxins and protects epithelial cells from the toxic stimulation [14, 15]. Meanwhile SIgA binding inhibits bacterial access and invasion to epithelial cells, SIgA binding to luminal bacteria also promote sampling of the bacteria to PPs, leading to induction of a strong response against the bacteria by B and T cells [33]. In general, SIgA exerts these functions without induction of deleterious inflammation at the site [25]. Although precise functions of SIgA that regulate host-microbiota homeostasis in the gut are not well defined, deficiency of SIgA in mice led to altered microbiome in the gut [17, 18] and to increased susceptibility to both pathogens and toxic reagents [17, 34].

### IgA influence on gut microbiome and health

There are several genetically modified mice which lack or have decreased SIgA in the gut lumen, and these models allow us to investigate the role of SIgA on gut homeostasis and host health. The effects of SIgA deficiency on models of infectious disease have been reviewed [14]. Here, we will focus on IgA influences on gut microbiota composition and gastrointestinal infection of bacteria. *Iga* ^−/−^ mice that lack the IgA switch region and the IgA constant region showed altered microbiota shift from newborn to adult [17]. *Iga* ^−/−^ mice showed increased and persistent colonization of Enterobacteriaceae family, which is predominant (> 75%) in newborn mice and decreased (< 1%) during the first 6 weeks [17]. It has been reported that the Enterobacteriaceae bacteria family is increased in patients with Crohn’s disease [2, 35], children with type 1 diabetes [36], and cirrhotic patients [21]. In addition, *Iga* ^−*/*−^ mice exhibited sustained intestinal inflammation and increased susceptibility to dextran sodium sulfate (DSS)-induced injury [17]. However, *Iga* ^−*/*−^ mice showed similar susceptibility to oral infection of *Citrobacter rodentium* infection compared with wild-type mice [37].

There are two other animal models that lack SIgA in the gut: pIgR-deficient and ‘joining’ chain (J chain)-deficient mice. These two mice cannot properly secret IgA or IgM into the gut lumen [14]. Decreased abundance of *Bifidobacterium* spp., which are known as beneficial bacteria in general, and increased abundance of *Helicobacter* spp. were observed in the cecum of the *Pigr* ^−*/*−^ mice [38]. Moreover, the *Pigr* ^−*/*−^ mice were susceptible to *Salmonella typhimurium* infection via the fecal–oral route [39] and more vulnerable to DSS-induced colitis than wild-type mice [34, 38]. However, *Pigr* ^−*/*−^ exhibited similar susceptibility to *Citrobacter rodentium* [37] and *Clostridium difficile* as compared with wild-type mice [40]. Immunized J chain-deficient mice were not protected from Cholera toxin [41], while they exhibited similar clearance of *Citrobacter rodentium* as wild-type mice [37]. There is no clear reason why these mice, lacking gut SIgA, showed normal susceptibility to several bacterial infection models. It is speculated that SIgA effects on simple bacterial infection model could be compensated by other type of immunoglobulins, such as SIgM and IgG, and/or antimicrobial peptides. Indeed, *Iga* ^−/−^ mice exhibited increased SIgM level in the gut [42] and mRNA of antimicrobial peptides were increased in the colon epithelium of *Pigr* ^−/−^ mice [38].

In humans, individuals with selective IgA deficiency have a tendency to develop infections of the gastrointestinal tract (e.g.,* Giardia* infections), celiac disease, and inflammatory bowel diseases [43]. Although selective IgA deficiency showed association with these diseases, 85–90% of IgA-deficient people are asymptomatic. However, there are few reports investigating the characteristics of gut microbiome of human IgA deficiency. Friman’s group demonstrated that IgA-deficient individuals more often had the genes involved in virulence of *Escherichia coli* in their rectal flora, although only several virulent factors of *E. coli* were examined [44]. Future studies that investigate IgA-deficiency on the human gut microbiome, will be important for this field of research.

## IgA-microbiota on liver diseases

### Gut microbiome-liver disease

Dietary factors including alcohol directly and indirectly influence the gut microbiota [45–48]. Intestinal bacterial overgrowth and bacterial dysbiosis after chronic alcohol consumption were observed in animals and human [47]. Intragastric alcohol feeding was associated with bacterial overgrowth in the large intestine as early as 1 week after feeding in mice [49]. Binge drinking of alcohol leads to elevation of blood endotoxin level. Alcohol and its metabolite acetaldehyde disrupts tight junction of epithelial cells and increase intestinal permeability [46]. Mice fed with high-fat diet exhibited altered gut microbiota composition [50, 51] and the change was independent from obesity [51], indicating high-fat diet itself influences gut microbiome composition. In addition, high-fat diet increased intestinal permeability through reduction of tight-junction proteins and induction of intestinal inflammation, leading to elevated blood endotoxin [45].

The mechanisms how altered gut microbiome contributes to development and progression of liver diseases were previously reviewed [20, 21, 47, 48, 52, 53]. Lipopolysaccharide (LPS), known as endotoxin, is a cell-wall component of gram-negative bacteria and interacts with Toll-like receptor 4 (TLR4). As mentioned above, increased level of circulating LPS was observed in patients with ALD and rodent models of ALD [54]. Increased endotoxin is also observed in alcoholic patients with minimal symptoms of ALD [55] and healthy subjects with single binge drinking [56]. In addition, the LPS levels correlate with disease severity [57, 58]. In mice, a LPS increase was observed both by acute binge gavage and chronic feeding with ethanol [59]. In addition to LPS, other bacterial components, such as bacterial 16S DNA and peptidoglycan which is a cell-wall component of gram-positive bacteria, were also increased in the circulating blood [56, 60]. In general, liver-resident macrophages, Kupffer cells, are tolerant to LPS-induced TLR4 activation and eliminate microbial components without inflammatory response. However, prolonged and excessive exposure to LPS can make Kupffer cells sensitive to LPS [20]. Moreover, ethanol-induced hepatocyte damage is associated with the liver macrophage activation through the damage associated molecular pattern production or signaling molecule-containing exosome production [61, 62]. Thus, sensitized Kupffer cells by ethanol-stimulated hepatocytes respond to gut-derived bacterial and fungal components and promote local inflammation in the liver. Other types of microbial products that are derived from the gut, such as bacterial DNA and cell-wall components, are also possible mediators of liver inflammation [20]. Recently, we found 1,3-β-glucan, which is a cell-wall component of fungi, is also increased in alcohol-fed mice and anti-*Saccharomyces cerevisiae* IgG antibody is significantly higher in alcoholic cirrhosis patients than in healthy individuals or viral cirrhosis patients [63]. Translocating fungi cell-wall component promoted IL-1β processing and local inflammation via its receptor, dectin-1, on liver macrophage cells [63].

Diet-induced endotoxemia is observed in rodent models of obesity and NAFLD/NASH [64–67]. In humans, NAFLD/NASH patients have higher level of systemic endotoxin when compared with healthy people [68, 69]. Infiltration of LPS into the liver has been shown to exacerbate liver injury in NAFLD/NASH mice, possibly via TLR4 activation [70]. Predisposition to obesity, which is a common factor attributed to NAFLD/NASH development, has been shown to be influenced by gut microbiota [71, 72]. Germ-free mice, which do not have any microorganisms in their body, are tolerant to high-fat diet-induced weight gain. By transplantation of gut microbiota from obese mice, but not from lean mice, germ-free mice gained more fat by high-fat diet [73]. That could be mediated by increased energy extraction from diet via altered microbiome. In addition, the production of fasting-induced adipocyte factor (Fiaf) is suppressed by enteric bacteria, resulting in increased activity of lipoprotein lipase and increased accumulation of triglycerides in the liver [21, 71, 72]. High-fat diet altered microbiota composition toward metabolizing dietary choline to methylamines, resulting in reduced level of plasma phosphatidylcholine [21, 74]. Choline-deficient diet, which also reduces phosphatidylcholine, is used to create rodent model of NASH. Reduced level of phosphatidylcholine, which is necessary for assembly of very-low-density lipoprotein (VLDL), results in triglyceride accumulation in the liver [21]. In addition, obese patients and children with NASH showed increased blood ethanol concentration [10, 75]. Increased ethanol produced by altered gut microbiome could possibly be a mediator of liver damage [21, 52].

### The role of IgA in NAFLD/NASH and ALD

IgA has been studied in the field of alcoholic liver disease for a long time [76]. Brown and Kloppel reviewed IgA levels in serum and the liver of ALD patients [29]. Systemic IgA levels are increased in patients with alcoholic liver disease [29] and in a rodent model of alcoholic liver disease [77]. In addition, liver IgA deposits appear specific for alcoholic liver disease, correlate with the amount of alcohol consumption, but not with serum IgA concentrations [78–80]. Moro-Sibilot and colleagues demonstrated that mouse and human liver contain IgA-secreting cells. Increased amount of IgA-secreting cells in mice fed with ethanol was diminished by inhibition of IgA-plasmablast egress from Peyer’s Patches with FTY720 that modulates sphingosine-1-phosphate receptor 1 and inhibits the egress of lymphocytes form secondary lymphoid organs, indicating that IgA deposits in the liver of ALD patients is attributed, at least partially, to increased entry of IgA-secreting cells from Peyer’s Patches to the liver [30].

SIgA levels in the gut of chronic liver diseases seems more important than systemic and liver IgA changes because SIgA regulates gut homeostasis. Studies that examined IgA levels in the gut of alcoholic patients and rodent models of ALD and NAFLD/NASH are summarized in Table 1. The number of IgA-secreting plasma cells in the intestinal lamina propria [30] and fecal IgA levels [77], which reflect the amount of colonic IgA, are reduced in experimental models of ethanol-induced liver disease of mice. These decreased SIgA and/or IgA-secreting cells in the intestine are reproducibly observed in rodent models of chronic alcohol feeding [81, 82], but not in rat model of single administration with ethanol [83–85]. Studies investigating intestinal IgA of patients with alcoholics are very limited [86–88]. Pelletier et al. observed decreased intestinal secretion of SIgA in patients with severe alcoholic cirrhosis when compared to patients with compensated alcoholic cirrhosis [86]. In contrast, Colombel et al. showed similar levels of jejunal SIgA secretion among alcoholics with cirrhosis, alcoholics without cirrhosis, and control subjects [87]. Maier and colleagues observed similar number of IgA-secreting cells in duodenum between alcoholics and controls, but a slightly decreased IgA amount in IgA-secreting cells of alcoholics with recent alcohol intake [88]. These conflicting reports may result from subjects in different stages of the disease or different methods of IgA measurement. Recently, we investigated the role of IgA in a mouse model of ethanol-induced liver disease by using *Iga* ^−*/*−^ mice. Unexpectedly, *Iga* ^−/−^ mice fed with ethanol showed similar degrees of liver injury (plasma ALT), hepatic triglyceride accumulation, and liver inflammation as compared with wile-type littermates [77]. Interestingly, we observed increased bacterial translocation to mesenteric lymph node in *Iga* ^−*/*−^ mice fed with control diet, but no further increase of the translocation in *Iga* ^−*/*−^ mice fed with ethanol. The result suggests that exposure of the liver of *Iga* ^−*/*−^ mice to gut-derived bacterial component is increased under physiological conditions and the increased translocation is further regulated by other factors than SIgA during disease. One possible reason why loss of IgA did not affect disease development is a compensation by SIgM. The *Iga* ^−/−^ mice fed with ethanol exhibited increased level of fecal IgM [77]. Further studies investigating compensatory roles of intestinal IgM and/or antimicrobial peptides are necessary.

**Table 1:** Changes in intestinal IgA associated with ALD and NAFLD/NASH in human and rodents

Human disease/animal model	Subjects	IgA (tissue)	IgA level	References
Human, alcoholics				
Alcoholic cirrhosis	Severe cirrhosis with jaundice, compensated cirrhosis, and control	Monomeric IgA and SIgA (perfusion fluid of proximal small intestine)	Severe cirrhosis < compensated cirrhosis = control	[86]
Alcoholics	Alcoholics with cirrhosis, alcoholics without cirrhosis, and control	SIgA (perfusion fluid of jejunum)	Alcoholics with/without cirrhosis = control	[87]
Alcoholics	Alcoholics with recent drink, alcoholics without recent drink, and control	Number of IgA plasma cells (duodenum)	Alcoholics = control	[88]
		Amount of IgA in IgA plasma cell (duodenum)	Alcoholics with recent drink <= alcoholics without recent drink and control	
Animal, ALD				
Wister rat, female	Intraperitoneal injection of ethanol (4 g/kg) for 30 min	IgA-positive cells (Peyer’s patch and lamina propria of small intestine)	EtOH > saline in lamina propria, EtOH = control in Peyer’s patch	[85]
Wister rat, female	Intraperitoneal injection of ethanol (4 g/kg) for 30 min, ± treatment with NOS inhibitor	IgA-positive cells (ileal lamina propria)	EtOH > EtOH + inhibitor = saline	[84]
Wister rat, male	Intraperitoneal injection of ethanol (4 g/kg) for 30 min, ± treatment with neuronal NOS inhibitor	SIgA (ileal extract)	EtOH > EtOH + inhibitor = saline	[83]
Wister rat, male	Lieber-DeCarli liquid diet for 4 weeks	IgA-positive cells (jejunal and colonic mucosa)	EtOH < control	[81]
C57BL/6 mouse, female	Lieber-DeCarli liquid diet for 8 weeks	SIgA (feces)	EtOH < control	[77]
C57BL/6-Ly5.1 mouse	Lieber-DeCarli liquid diet for 10 days + binge (5 g/kg) for 9 h	IgA-positive plasma cells (small intestinal lamina propria)	EtOH < control	[30]
C57BL/6 mouse	20% w/v ethanol in drinking water for 10-12 weeks	SIgA (contents of small and large intestine)	EtOH < control	[82]
Animal, NAFLD/NASH				
Sprague–Dawley rat, female	High-fat diet (88% normal + 10% lard + 2% cholesterol) for 8-12 weeks	SIgA (small intestinal mucosa)	HF = < control in 8 weeks, HF < control at 12 weeks	[95]
Sprague–Dawley rat, male	High-fat diet (30% w/w beef tallow) for 3 weeks, ± supplied with curcumin	SIgA (feces)	HF + curcumin > HF > LF + curcumin = LF	[92]
Sprague–Dawley rat, male	High-fat diet (30% w/w beef tallow) for 3 weeks, ± supplied with sericin	SIgA (feces and colon)	HF + sericin > HF = LF = LF + sericin in feces	[93]
			HF + sericin = LF + sericin > HF = LF	
Sprague–Dawley rat	High-fat diet (60% fat energy) for 8 weeks, ± supplied with polyphenols	SIgA (feces)	HF + polyphenols > HF = LF	[96]
C57BL/6 mouse, male	Methionine-choline-deficient diet for 3 weeks, ± supplied with fructo-oligosaccharide	SIgA (feces), IgA-positive cells (ileum and colon)	MCD < control < MCD + fructo-origosaccharide (SIgA and IgA-positive cells)	[94]

*SIgA* secretory IgA, *EtOH* ethanol, *HF* high-fat diet, *LF* low-fat diet, *MCD* methionine-choline-deficient diet, *NOS* nitric oxide synthase

Unlike the case of ALD, reports that investigated an association between serum IgA level and NAFLD/NASH are limited [89–91]. Tomita et al. showed that serum IgA concentration is increased in severe-NASH patients when compared with early-stage NASH patients, although IgA concentrations were comparable among control, simple steatosis, NAFLD, and early-stage NASH [89]. McPherson’s group showed that serum IgA was elevated in NASH patients as compared with simple steatosis patients [90]. In these studies, serum IgA levels were positively associated with severity of liver fibrosis [89–91]. However, associations between increased serum IgA and advanced stage are also observed in other types of chronic liver diseases. Thus, increased systemic IgA levels in NAFLD/NASH patients may not be a specific marker for the disease progression. Although there is no study that investigated intestinal or fecal IgA levels of NAFLD/NASH patients, several studies using rodent models of NAFLD/NASH showed altered intestinal IgA secretion [92–96]. In the study of Matsumoto’s group, mice with NASH induced by methionine-choline-deficient diet, exhibited decreased fecal IgA and IgA-positive cells in ileal and colonic tissues [94]. In addition, Li et al. showed decreased IgA in small intestinal mucosa of rats fed with high-fat diet, and a negative correlation between the intestinal IgA levels and systemic endotoxin levels [95]. However, similar IgA levels of feces between high-fat and control groups or increased fecal IgA in high-fat group were observed in several studies [92, 93, 96]. Different feeding protocol might explain these results. In the study from Li’s group, decreased SIgA in the small intestine was not observe in 8-week feeding group, but observed in 12-week feeding group [95]. Although the mechanisms how increased systemic IgA and/or reduced intestinal SIgA are involved in disease development and progression are not clear yet, some gut dysbiosis and bacterial overgrowth observed in ALD and NAFLD/NASH might contribute to decreased SIgA levels in the gut.

### The role of SIgA on gut microbiome in NAFLD/NASH and ALD

The study of gut SIgA functions in liver diseases is the field that started recently, and SIgA behavior in the gut of human alcoholic patients has not been intensively investigated yet. However, several studies revealed shared features of gut microbiome changes between SIgA-deficient mice and human patients with alcoholic liver disease. As mentioned above, *Iga* ^−*/*−^ mice exhibited increased colonization of Enterobacteriaceae family (in phylum Proteobacteria) [17]. Increased abundance of phylum Proteobacteria is a signature of intestinal dysbiosis associated with metabolic and inflammatory diseases [97]. Moon et al. found dichotomous fecal IgA levels among wild-type mice in the same facility and the IgA-low phenotype was driven by transmissible fecal bacteria [98]. This IgA-low phenotype of mice was associated with enriched Enterobacteriaceae family. Although increased Enterobacteriaceae family was not observed in IgA-low mice from a different facility in the same study, the phylum Proteobacteria was increased in the mice [98]. These results indicate decreased SIgA may drive the expansion of Enterobacteriaceae family (or phylum Proteobacteria) in the gut of chronic liver diseases, such as ALD and NAFLD/NASH. Increased members of the Enterobacteriaceae family or phylum Proteobacteria is also observed in chronic liver diseases [21]. Zhu et al. and colleagues revealed that abundance of *Escherichia* spp. (in Enterobacteriaceae family) and Enterobacteriaceae family are increased in stool of children with NASH when compared with healthy children or children with obesity [10]. Increased *Gammmaproteobacteria* class, which includes Enterobacteriaceae family, was also observed in children with NAFLD when compared with healthy children in a different study [99]. A study in which gut microbiota composition were compared between 53 NAFLD patients and 32 healthy subjects showed increased *Escherichia* spp. in NAFLD patients [100]. Increased abundance of phylum Proteobacteria and/or Enterobacteriaceae family were reproduced in rodent models of NAFLD [101–104]. However, the enrichment of Enterobacteriaceae and *Escherichia* spp. was not reproduced in human studies [105, 106]. Taken together, decreased amount of SIgA in the gut lumen, which was observed in rats fed with high-fat diet [95], may contribute to expansion of phylum Proteobacteria and/or Enterobacteriaceae family of bacteria and disease pathogenesis.

The prominent feature of dysbiosis observed in alcoholic patients and rodent model of alcoholic liver disease is characterized by a decreased phylum Firmicutes and lower amount of *Lactobacillus* spp. (in phylum Firmicutes) [49, 107–110]. Probiotic treatment with *Lactobacillus* spp. and *Bifidobacterium* spp. improved bacterial dysbiosis and ameliorated liver injury and/or inflammation in both humans and animal models (previously reviewed by Bluemel et al. [111]). In addition to these bacteria, alcoholic patients with dysbiosis showed a higher relative abundance of *Gammmaproteobacteria* class (in phylum Proteobacteria) than alcoholic patients without dysbiosis [112]. Relative abundance of Enterobacteriaceae family was higher in the stool of patients with alcohol hepatitis than alcoholic patients without hepatitis [113]. Increased Enterobacteriaceae is reproducibly observed in human cirrhotic patients [9, 11, 114]. Bajaj et al. proposed the cirrhosis dysbiosis ratio (CDR). The ratio of “good” bacteria, such as *Lactobacillus* spp., against potentially “bad” bacteria, such as Enterobacteriaceae was lower in advanced cirrhotic patients as compared with that in compensated-cirrhotic patients [58]. Especially, cirrhotic patients with alcoholic etiology showed increased Enterobacteriaceae family [58]. Although these facts suggest a possible link between reduced SIgA and development of the disease, studies investing direct effects of SIgA on the diseases are limited. Further studies investigating the change of gut microbiome and disease status in SIgA-deficient mice are required.

## Fc receptor alpha I and IgA on liver diseases

In addition to the SIgA roles in the intestinal lumen, it has been suggested that serum and lamina propria IgA play a role in mucosal immunity via binding to a transmembrane receptor, Fc receptor alpha I (FcαRI) [115]. Functions of FcαRI are previously reviewed [115, 116]. FcαRI is expressed on cells of the myeloid lineage, including neutrophils, eosinophils, and Kupffer cells [116]. In contrast to non-inflammatory functions of SIgA, serum monomeric IgA and dimeric IgA (dIgA) in the lamina propria can induce several pro-inflammatory functions via the binding to FcαRI [115]. Cross-linking of FcαRI by IgA immune complexes (e.g., IgA-coated bacterial cells) mediates the production of reactive oxygen species and lymphokine from neutrophils and promotes phagocytosis by neutrophils and Kupffer cells [115, 117]. However, studies investigating this topic are lacking, because mice do not have an equivalent receptor of the human FcαRI [118].

Egmond’s group revealed serum IgA to eliminate bacteria invading the liver, by using mice with a transgene that is introduced with a cosmid clone containing the human FcαRI gene [117]. Kupffer cells that express the human FcαRI effectively phagocytosed bacteria coated with serum IgA, but not with SIgA. In contrast, the number of phagocytosed bacteria in the liver of non-transgenic mice were only 20% of that of the transgenic mice [117]. In addition, neutrophils isolated from healthy human donors responded to serum IgA and produce leukotriene B4 (LTB4), leading to further recruitment of neutrophils [119]. Mouse neutrophils that express human FcαRI phagocytosed IgA-coated bacteria more than BSA-coated bacteria. In the colon of patients with ulcerative colitis, a number of FcαRI-expressing neutrophils infiltrated the lamina propria and phagocytosed IgA-complexes. In contrast, few neutrophils were observed in the colon of healthy subjects [119]. These results indicate that IgA opsonizes bacteria and promotes effective phagocytosis of and elimination of the invading bacteria via binding to FcαRI on phagocytes. Thus, serum IgA and lamina propria dIgA can work as second line barrier against translocated bacteria from the gut lumen to lamina propria, portal blood, and the liver which is observed in patients with ALD and NAFLD/NASH. However, excessive activation of this function potentially can lead to local inflammation. There is no study investigating the role and expression of FcαRI in the liver and/or gut of patients with ALD and NAFLD/NASH and of animal models. These studies are necessary to understand the precise role of IgA on pathogenesis of diseases.

## Future directions

The intestinal microbiome has been suggested to contribute to the development and progression of ALD and NAFLD/NASH. However, precise mechanisms how the gut microbiome contributes to the disease pathogenesis and how the development of diseases disrupts gut homeostasis are not fully understood. Some features of altered gut microbiome, such as increased phylum Proteobacteria and Enterobacteriaceae family of bacteria, are shared between IgA-deficient animals and patients with and animal models of ALD and NAFLD/NASH. In addition, chronic feeding of alcohol and an obesogenic diet causing NAFLD/NASH led to decreased intestinal production of IgA in rodents. These facts imply the potential role of SIgA on the liver diseases. However, there are limited studies that investigate SIgA change in patients and reveal direct influence of IgA-deficiency on development of ALD, NAFLD or NASH. These studies are necessary in the future.

In addition to SIgA in the gut homeostasis, the role of circulating IgA, which is increased in ALD and NASH with cirrhosis, also requires to be investigated in future studies. It has been suggested that FcαRI on Kupffer cells and neutrophils contribute to elimination of gut-derived bacteria and its products via activating phagocytosis. However, it has been difficult to investigate the role of IgA-FcαRI in development of the liver diseases because mice lack equivalent receptor of human FcαRI. To apply the transgenic mice introduced with the human FcαRI gene to investigate the role could be a solution of the problem.
